# Newborn Screening for Krabbe Disease—Illinois Experience: Role of Psychosine in Diagnosis of the Disease

**DOI:** 10.3390/ijns7020024

**Published:** 2021-05-09

**Authors:** Khaja Basheeruddin, Rong Shao, Fran Balster, Pearlie Gardley, Laura Ashbaugh

**Affiliations:** 1 Newborn Screening Laboratory, Illinois Department of Public Health, Chicago, IL 60612, USA; Rong.Shao@illinois.gov (R.S.); Fran.Balster@illinois.gov (F.B.); Pearlie.Gardley@illinois.gov (P.G.); 2 Office of Health Promotion, Illinois Department of Public Health, Springfield, IL 62671, USA; Laura.ashbaugh@illinois.gov

**Keywords:** newborn screening, dried blood spot, galactocerebrosidase, Krabbe disease, psychosine, lysosomal enzymes

## Abstract

Population-based newborn screening for Krabbe disease was initiated by measurement of galactocerebrosidase (GALC) activity in the state of Illinois in December 2017. Due to the poor specificity of GALC for the diagnosis of Krabbe disease, second-tier testing services were provided to reduce the false positive rates for disease monitoring. Using ultra-pressure liquid chromatography coupled to mass spectrometry assay, a total of 497,147 newborns were screened. In total, 288 infants’ specimens (0.06%) having reduced GALC activity were sent out for second-tier testing to a reference laboratory. All newborns’ reduced GALC specimens were tested for psychosine levels, the presence of a 30-kb deletion and GALC sequencing. The results showed that two infants had elevated psychosine levels (10 and 35 nM) and were referred immediately for evaluation and treatment for Infantile Krabbe disease, and six infants had intermediate PSY levels (≥2 to 5 nM) and are under observation as suspected candidates for late-onset Krabbe disease. In addition, 178 infants had pseudodeficiency alleles, all having psychosine levels < 2.0 nM. Our data show that a high percentage of reduced GALC activity (62%) was due to the presence of pseudodeficiency alleles in the GALC gene. In conclusion, incorporation of psychosine measurements can identify infants with infantile Krabbe disease and probable late-onset Krabbe infants. Furthermore, Krabbe disease screening can be achieved at public health laboratories, and infants with infantile Krabbe disease can be diagnosed in timely manner for better outcome.

## 1. Introduction

The State of Illinois initiated newborn screening (NBS) for Krabbe disease (KD; globoid cell leukodystrophy; OMIM 245200) in December 2017 by measuring galactocerebrosidase (GALC; EC 3.2.1.46) activity in dried blood spots (DBSs). The statewide screening program includes the measurement of seven lysosomal enzymes to screen for lysosomal storage diseases (LSDs) using tandem mass spectrometry [1,2,3]. Pompe disease and Mucopolysaccharidosis I (MPS I) are the only two lysosomal diseases included on the federal Recommended Uniform Newborn Screening Panel (RUSP) by the Advisory Committee on Heritable Disorders in Newborns and Children of the Department of Health and Human Services. In addition to these recommended LSDs, however, several states have started mandatory statewide testing for KD, initiated primarily as a result of parent advocacy [4,5].

Infants with GALC deficiency have defective myelin turnover and may develop the infantile form of KD (IKD) [6]. Such infants appear to be normal at birth, and during their first three to six months of life, they show signs of irritability and feeding difficulties and develop rapid neurological complications, resulting in death during infancy or childhood [7]. Late onset for KD includes patients with onset at an age of 13 months to 10 years and is characterized mainly by abnormal gait due to spastic paraparesis. Decreased GALC activity detected in NBS is not a good predictor of KD with confidence due to low specificity [4,8]. The non-specific reduction in enzyme activity due to the presence of many known pseudodeficiency variants in vitro using the current artificial substrates is common, with no clinical evidence of disease. These variants, if inherited as homozygous mutations or in trans with a disease-causing mutation, do not cause disease. However, the presence of some of these mutations in cis with another mutation can make the allele disease causing. In addition, variants of uncertain significance (VUS) also cause decreased GALC activity. These variants make referring infants for follow-up based on screening alone more challenging [8,9]. Although GALC is a reasonable first-tier test, it does not have acceptable specificity on its own; to increase specificity, there is accumulating evidence that second-tier testing for KD should include measurement of psychosine (PSY; galactosyl-sphingosine) levels and gene sequencing [10]. PSY is one of the substrates for GALC, and its concentration in DBS correlates with IKD [8,11]. Genetic testing results indicative of IKD include detection of a homozygous 30-kb deletion or two severe pathogenic mutations in trans [7]. In addition, IKD cannot be excluded if gene sequencing reveals the presence of only one pathogenic mutation because of uncertainty of missing other possible disease-causing mutations in GALC exon sequencing.

A vast amount of data show that in those specimens that are screened as positive for GALC, coupling the assay with PSY and sequencing results will improve the specificity of IKD diagnosis [4,5,10]. Recent studies by Guenzel et al. (2020) showed that infants suspected of having IKD disease have elevated PSY levels, whereas intermediate PSY levels warrant an evaluation for late-onset variants [11]. Considering PSY being an important marker for identifying IKD presumptive babies, Herbst et al. (2020) conducted studies using a common set of DBS calibrators. PSY levels in reference and participating laboratories adopted similar reference ranges and could identify presumptive IKD infants [12]. Escolar and colleagues reported an improved outcome by performing human stem cell transplantation (HSCT) in IKD-diagnosed infants during their pre-symptomatic stage as compared to infants’ treatment after symptoms began [8]. As a result, current recommendations are to treat IKD-diagnosed infants in the pre-symptomatic stage, preferably during their first month of life [10]. 

The purpose of this paper was to describe three years’ Krabbe NBS experience in a statewide laboratory program at the Illinois Department of Public Health (IDPH) and to show how second-tier testing results were utilized for diagnosis of IKD. The evidence presented shows that Krabbe screening can be achieved at the state public health level. In addition, this study shows that PSY and 30-kb deletion results could improve diagnosis in a timely manner and provide the opportunity for early treatment and better outcomes for infants in the pre-symptomatic stage.

## 2. Methods

Testing was initiated by incubating a 3.2-mm DBS punch in 96-well plates in the presence of six enzyme substrates and their respective internal standards. In addition to buffer and detergent, inhibitors to block non-specific enzyme reactions associated with GAA, GLA and IDUA were also included in the cocktail. A second 3.2-mm punch from the same DBS was also incubated for 17 h at 37 °C with the substrate and internal standard for I2S along with required salts and processed. All substrates and internal standards were purchased from Perkin Elmer, Waltham, Massachusetts. Enzyme reactions were quenched by adding 100 µL acetonitrile for the 6-plex 96-well plate and 200 µL acetonitrile for the 1-plex 96-well plate. An aliquot from I2S plates was transferred from the samples in the I2S plates to the corresponding samples in the 6-plex plate. Ultraperformance liquid chromatography coupled to tandem mass spectrometry (UPLC-MS-MS) was used to measure each enzyme activity without liquid extraction. The enzyme activities were expressed as % batch median consisting of samples from one or more plates analyzed on the same day [1]. 

An initial screen for GALC was performed in a single punch, and if the GALC activity was less than or equal to 16% of the batch median, the analysis was repeated in five replications. A positive GALC screen was defined as a mean of repeated activity of less than or equal to 13% of the batch median. At this point, a screen-positive GALC specimen was sent out to a reference laboratory (Mayo Clinic Laboratories, Rochester, MN/PerkinElmer Genetics, Inc., Pittsburg, PA, USA) for second-tier testing. The second-tier testing provided PSY level and genetic analysis data consisting of screening for the 30-kb deletion followed by GALC gene sequencing. The sent-out specimen status stayed as “pending”, as shown in “Call-Out” column, until GALC gene sequencing results were received (Table 1). Abnormal PSY level (greater than or equal to 8.7 nM; PerkinElmer Genetics, Inc., Pittsburg, PA, USA) and/or detection of a homozygous 30-kb deletion triggered immediate referral to a consultant/specialized center for the purpose of evaluation for HSCT as shown in the “Follow-Up” column. In the case of negative PSY and 0–1 copies of the 30-kb deletion, the specimen result was not called out (N/A in “Follow-Up” column) until sequencing results were released by the reference laboratory. A detailed call-out protocol is described in Table 1. The 30-kb deletion assay was performed as a genotype assay for detecting wild-type and mutant alleles. In addition, the next-generation sequencing assay was performed using a gene panel to determine the genomic variations present in the GALC gene. The 30-kb deletion was confirmed in the sequencing analysis using break point probes.

**Table 1:** GALC screening, analytical and post-analytical workflow and initial reporting for KD at IDPH.

Screening Lab	Initial GALC * Activity	Repeat GALC Screen	Repeat GALC * Activity	Send Out Lab	Psychosine (nM) **	30-kb Deletion	Seq Result	Call-Out	Follow-Up
IDPH	>16	No		N/A ***	N/A	N/A	N/A	Negative	N/A
IDPH	≤16	Yes	≤16–>13	N/A	N/A	N/A	N/A	Negative	N/A
				Yes	≥8.7	ND ^▪^/H ^□^/HET ^◊^	Waiting	Positive	Referral ASAP
IDPH	≤16	Yes	≤13	Yes	<8.7	ND/HET	Waiting	Pending	N/A
				Yes	<8.7	ND/HET	Received	See Table 2	See Table 2

Abbreviations: * galactocerebrosidase activity expressed as % batch median; ** PSY normal range < 8.7 nM; *** not applicable; ^▪^ not detected; ^□^ homozygous; ^◊^ heterozygous.

**Table 2:** Assignment of diagnosis after receiving second-tier testing results for KD (n = 288).

GALC * Activity	*N*	% Send Out	PSY Levels (nM) **		Mutations Detected	Diagnosis	Follow Up
			<2 nM	>2–<3 nM			
≤13%	178	62	178	0	Pseudodeficiency alleles	Negative ^1^	No
≤13%	35	12	30	5	VUS ^□^	VUS ^2^	No
≤13%	67	23	60	7	One pathogenic allele or heterozygous 30-kb deletion	Carrier ^3^	No
<12%	6	2	See Table 3		Two pathogenic mutations	Late onset	Yes
<11%	2	0.7	See Table 3		Two pathogenic mutations	IKD ^#^	Yes

Abbreviations: * galactocerebrosidase activity expressed as % batch median; ** PSY normal range < 8.7 nM; ^□^ variants of uncertain significance; ^#^ infantile KD; % send out: Fraction of all samples sent for second-tier testing. Total number of newborns screened was 494,147. ^1^ Infants with only pseudodeficiency allele/s. ^2^ Most of the infants in this category had VUS and two pseudodeficiency alleles. ^3^ Infants in this category had one pathogenic variant, or one VUS, with or without pseudodeficiency alleles classified as carriers.

**Table 3:** PSY levels and mutations detected in late-onset suspected and IKD cases.

Suspected Late on-Set Krabbe (Age ^#^)	PSY (nM)	Mutations *
Case 1 (38)	6	Heterozygous pathogenic c1671G>A; VUS c.235C>T; PD C.1685T>C
Case 2 (32)	2	Heterozygous pathogenic c.430delA and c.1901T>C; 2 heterozygous PD c.550C>T; c.1685T>C
Case 3 (28)	3	Homozygous pathogenic c.349A>G
Case 4 (13)	3	Heterozygous VUS c.334A>G; c.977C>T; heterozygous PD c.1685T>C
Case 5 (12)	3	Heterozygous VUS c.334A>G; c.977C>T; heterozygous PD c.1685T>C
Case 6 (8)	5	Homozygous pathogenic c.956A>G
IKD- transplanted		
Case 1 (32)	10	Heterozygous pathogenic alleles: c.1171_1175het_delCATTCinsA and c.749T>C
Case 2 (25)	35	Heterozygous likely pathogenic alleles: c.1723_1724insT and c.1913G>T

^#^ months; * detected by next-generation sequencing (exons), confirmed by Sanger sequencing analysis and variants classified based on ACMG standard criteria (PMID:25741868) and recent clinical guidelines (https://www.clinicalgenome.org/working-groups/sequence-variant-interpretation/) and evaluated by gnomD, HGMD and ClinVar.

Diagnostic results due to the presence of a single pathogenic variant or variant(s) of uncertain significance (VUS) and PSY level were communicated to submitters by the Illinois Department of Public Health Genetics Program staff. Alternatively, a negative diagnostic result was communicated to submitters only if pseudodeficiency allele(s) were detected. For presumptive IKD cases, the staff made sure that the infant is seen by a specialist immediately and provided all necessary results to the specialist for the first appointment. 

## 3. Results

The IDPH screened a total of 494,147 newborns between 8 December 2017 and 31 December 2020. A total of 838 newborns had less than or equal to 16% of the batch median GALC activity and testing was repeated for the confirmation assay. The repeat analysis yielded 288 positive specimens having less than or equal to 13% batch median GALC activity that were sent out to the reference laboratory for second-tier testing. In a few cases, the specimens were called out as “Invalid” due to variation in spot activity (>25% CV) in the repeat analysis and a second specimen was requested. Table 2 describes the GALC activity of the sent-out specimens with their second-tier testing data, showing PSY levels, molecular results and how specimens were reported. The PSY level in newborns plays an important role [11]. All newborns with pseudodeficiency (62% of sent-out specimens) alleles had PSY levels of less than 2 nM. There were a number of cases having VUS (12%). Infants having a single pathogenic mutation or one copy of the 30-kb deletion (heterozygous) were identified as carriers (23%) and follow-up was not recommended for both VUS- and carrier-assigned categories. Although, there were a few cases where the PSY level was more than 2 but less than 3 nM (Table 2). 

Table 3 summarizes the PSY levels and mutations detected in newborns under observation for suspected late-onset KD, and two infants were transplanted due to the presence of disease-causing mutations with high PSY levels. The IKD-diagnosed infants had PSY levels of 10 and 35 nM, whereas the late-onset diagnosed infants had an intermediate PSY level [11] but a genotype previously observed in association with late-onset disease. Two infants were followed up immediately for IKD and were treated. In addition, six more infants suspected of probable late-onset KD will be followed up with a specialist until they turn thirteen for early signs of the disease. An infant with probable late-onset KD may be identified by the presence of one pathogenic allele (30-kb deletion or any other) or one VUS (which could be pathogenic) with or without pseudodeficiency alleles and PSY above 2 nM. The remaining infants were categorized as VUS, carriers and negative, as shown in Table 2. For the negative category, all specimens had negative/normal PSY levels (<2.0 nM), and the GALC gene sequencing revealed the presence of pseudodeficiency alleles. Specimens having a heterozygous 30-kb deletion or a heterozygous pathogenic mutation with PSY lower than 2 nM were categorized as carriers. There were a few specimens with a carrier or VUS result from molecular testing having PSY levels more than 2 nM. After follow-up/specialist visit, these cases were closed based on having a normal PSY level.

The genetic testing for the specimens not associated with IKD revealed the presence of pseudodeficiency alleles (62%) and VUS (12%). Infants having a single pathogenic mutation or one copy of a 30-kb deletion (heterozygous) were identified as carriers and follow-up was not recommended (23%). However, an intermediate group of infants who had mildly elevated PSY (>2–<8.7 nM) in the newborn screen specimen and had two pathogenic mutations were followed up regularly due to high risk for late-onset disease. Additionally, included in this group would be infants with a single mutation if there were a persistent PSY elevation.

## 4. Discussion

An increasing number of public health laboratories are screening for LSDs, mostly for MPS I and Pompe. However, in addition to Illinois, seven states’ laboratories are screening for KD (NY, NJ, OH, TN, MO, KY and IN). The driving force for renewed interest in LSD screening is the availability and reliability of new testing methods for detection and an increasing number of early interventions. Over a period of three years, screening of newborns at birth for LSDs, including KD, in Illinois led to earlier intervention and improved outcome for two infants diagnosed with IKD. Six more infants were diagnosed as suspected candidates of late-onset KD and are under close observation. Out of this group, the oldest child at the age of 3 years was not showing any signs of the disease. Of the 494,147 newborns screened for seven LSDs in Illinois over the period of three years, the data for KD disease showed that for every 3000 specimens screened, six specimens or 0.2% specimens screened were repeated for confirmatory testing. Overall, for every 2000 specimens screened, one specimen (0.05%) was sent out for second-tier testing as compared to 0.02% in other NBS programs [4].

Krabbe screening proved to be more challenging than screening for some other LSDs due to the absence of a clear relationship between the measured enzyme activity and phenotype or disease status. In addition, GALC-specific enzyme activity is low compared to other lysosomal enzymes [13,14] and, coupled with the urgency for identifying presumptive infantile cases in a short time, makes Krabbe screening more challenging. With these uncertainties, reporting GALC screening results directly to health care providers would have resulted in many more infants being subjected to follow-up testing and psychological stress on many more families. For this reason, the IDPH decided to hold screening results until second-tier testing results consisting of PSY, and 30-kb deletion and gene sequencing were available to report. The IDPH was able to identify IKD cases with more confidence based on PSY results within 8–10 days of receiving date of the specimens (data not shown). Two infants were confirmed by this protocol and were able to receive treatment (HSCT), one within four weeks and the second infant within seven weeks of their life, and both are doing well at 14 and 12 months of age, respectively, with mild developmental delays [15].

The overall incidence of IKD in Illinois was found to be 1 in 250,000, which is higher than that reported in other states [4]. This rate can change as more newborns are tested. PSY and 30-kb deletion results were generally received from the reference laboratory within 2–3 days. As a result, the screened positive GALC specimens could be immediately referred for IKD follow-up. Only 22 out of 288 screened positive specimens were heterozygous for the 30-kb deletion and none were homozygous. The deletion was not detected in either of the IKD-confirmed cases. This result is different from the Missouri Krabbe screening results, where more homozygous 30-kb deletion cases were detected [4]. High PSY levels in IKD cases suggest that relying on 30-kb deletion detection as a second-tier test for IKD may provide inaccurate results (Table 2). However, our data confirm that PSY is a good indicator for confirming IKD status and should be incorporated in screening programs for KD.

Although an initial GALC screen specimen having less than a <16% batch median is repeated in five spots to minimize assay imprecision near the screen cut-off, there is ample evidence that the normalization of GALC activity with other lysosomal enzymes will be more informative, perhaps due to normalizing differential cell count in DBS and other factors [4,16]. The normalization with respect to two RUSP-recommended and six lysosomal enzymes significantly reduced screen-positive call-out to 0.009% and 0.006%, respectively, compared to 0.06% without normalization (data not shown). It was also noted that normalization using six enzymes’, or two enzymes’ activities generates similar results (data not shown).

To reduce the referral rate for either second-tier testing or follow-up, a post-analytical interpretive tool based on multivariate pattern recognition software developed by Mayo Clinic (collaborative laboratory interpretive reports (CLIR), https://clir.mayo.edu) is available for measuring the likelihood of disease using percentile rankings. Our data show a higher number of positive GALC specimens to be sent out for second-tier tests. When positive GALC specimens are tested for PSY, the resulting PPV can be improved significantly from 2.8% to 40% (Table 4). The PPV reached 100% if PSY and 30-kb deletion and sequencing results were incorporated, with two infants identified as having IKD due to high PSY levels and the presence of pathogenic mutations, and six infants were labeled as suspected late onset due to moderate levels of PSY with certain mutations. To date, no false negative case has been reported. Six infants were identified for observation as having probable late-onset disease status based on sequencing results. The PSY levels in IKD cases were in the positive range > 8.7 nM (PerkinElmer Genetics, Inc., cut-off) whereas the late-onset KD infants’ levels were below 8.7 nM, suggesting that the gene sequence information is required to see if disease-causing mutation(s) can be identified. In addition, the PSY levels in the range of <2.0 nM consist of specimens having one pathogenic mutation (carrier), pseudodeficiency or most of the VUS mutations, except for a few specimens having a PSY value between 2 and 3 nM. Our data also show that late-onset specimens have PSY levels in an intermediate range of 2–8.7 nM. This observation agrees with recently published data [11]. In addition, our results also confirmed consensus guidelines for the diagnosis and initial referral for treatment of IKD suggested by Kwon et al. (2018) [10]. This observation highlights the importance of the utilization of a common set of DBS calibrators among reference laboratories such that similar PSY values for the limited number of presumed IKD specimens can be reported [12]. We are in the process of updating the PSY cut-off (to 10 nM) to reflect an agreed value among the testing/reference laboratories. In addition, we are planning to not test sequencing results for a PSY level less than 1.5 nM. The workflow in the IDPH laboratory of holding Krabbe screening results until sequencing results are received is beneficial for families. As a result, Krabbe screening in Illinois had a low impact on families, and affected infants presumed to have IKD were detected and treated in a timely manner [17,18].

## 5. Summary

Newborn screening for KD in Illinois using UPLC-MS-MS technology has been ongoing for over three years, providing GALC activity, PSY, 30-kb deletion and GALC sequencing results. The program has been beneficial in identifying infants affected with infantile and probable late-onset forms of the disease. These affected cases were referred to specialists and led to successful treatment of two infants with IKD by HSCT. In conclusion, Krabbe newborn screening can be successfully integrated into a state newborn screening program, leading to an improved outcome for infants affected by this disorder.

## Figures and Tables

**Table 4:** Results from 2 years of full population newborn dried blood spot screening for KD in Illinois.

	n	% Total Screened	PPV *	FPR **
Total specimens screened	494,147			
Screen positive specimens	288	0.06		
Pseudodeficiency allele/s (negative)	178	0.036		
VUS ^#^ allele/s	35	0.007		
Carrier	67	0.013		
Diagnosed (2 IKD ^•^ and 6 LOKD ^••^)	8	0.001	2.8%	56/100,000
Expected screen positive after 2nd tier test/s				
PSY (>2.0)	20		40%	2.4/100,000
Sequencing results	8		100%	

Note: * positive predictive value calculated; ** false positive rate calculated; ^#^ variants of uncertain significance; ^•^ infantile Krabbe disease; ^••^ late-onset Krabbe disease.

## Abbreviations

GALC	Galactocerebrosidase
KD	Krabbe disease
LSD	Lysosomal storage disease
IKD	Infantile Krabbe disease
HSCT	Human stem cell transplantation
PSY	Galactosyl-sphingosine, psychosine
VUS	Variant of uncertain significance
IDPH	Illinois Department of Public Health
